# Cervical Spondylosis Diagnosis Based on Convolutional Neural Network with X-ray Images

**DOI:** 10.3390/s24113428

**Published:** 2024-05-26

**Authors:** Yang Xie, Yali Nie, Jan Lundgren, Mingliang Yang, Yuxuan Zhang, Zhenbo Chen

**Affiliations:** 1 Department of Medical Imaging, China Rehabilitation Research Center and Capital Medical University School of Rehabilitation Medicine, Beijing 100068, China; ssyangxie@gmail.com; 2 Department of Electronics Design, Mid Sweden University, 85170 Sundsvall, Sweden; yali.nie@miun.se (Y.N.); jan.lundgren@miun.se (J.L.); yuxuan.zhang@miun.se (Y.Z.); 3 Department of Spinal and Neural Function Reconstruction, China Rehabilitation Research Center and Capital Medical University School of Rehabilitation Medicine, Beijing 100068, China; chinayml3@163.com

**Keywords:** cervical spondylosis, X-ray classification, multi-label, deep learning

## Abstract

The increase in Cervical Spondylosis cases and the expansion of the affected demographic to younger patients have escalated the demand for X-ray screening. Challenges include variability in imaging technology, differences in equipment specifications, and the diverse experience levels of clinicians, which collectively hinder diagnostic accuracy. In response, a deep learning approach utilizing a ResNet-34 convolutional neural network has been developed. This model, trained on a comprehensive dataset of 1235 cervical spine X-ray images representing a wide range of projection angles, aims to mitigate these issues by providing a robust tool for diagnosis. Validation of the model was performed on an independent set of 136 X-ray images, also varied in projection angles, to ensure its efficacy across diverse clinical scenarios. The model achieved a classification accuracy of 89.7%, significantly outperforming the traditional manual diagnostic approach, which has an accuracy of 68.3%. This advancement demonstrates the viability of deep learning models to not only complement but enhance the diagnostic capabilities of clinicians in identifying Cervical Spondylosis, offering a promising avenue for improving diagnostic accuracy and efficiency in clinical settings.

## 1. Introduction

Cervical Spondylosis (CS) is a chronic degenerative condition of the cervical spine, manifesting through various clinical symptoms such as neck pain, stiffness, radiating pain, headaches, and the potential onset of neurological dysfunction [1]. Significantly, neck pain was identified as the fourth leading cause of disability-adjusted life years (DALYs) in 2015, highlighting the global impact of CS [2]. Epidemiologically, the prevalence of CS varies by age, with rates of 12.4% in individuals aged 18–29, escalating to 100% in those over 70 years, indicating a widespread and age-related increase in incidence. Early stages of CS might not disrupt daily life significantly, but as the condition progresses, symptoms worsen, leading to increased treatment complexity and healthcare costs, thereby underscoring the importance of early detection [3].

The diagnostic process for CS primarily employs imaging modalities such as Computed Tomography (CT), Magnetic Resonance Imaging (MRI), and X-ray [4], with X-ray screening being the preferred method due to its accessibility, low radiation exposure, and cost-effectiveness [5]. Despite its advantages, X-ray diagnosis of CS is hindered by its lower accuracy and the reliance on the subjective judgment and experience of the interpreting physician [6]. This situation calls for improved diagnostic methodologies to overcome these limitations [7].

Recent advancements in artificial intelligence (AI), particularly in machine learning (ML) and deep learning (DL), have made significant strides in medical diagnostics, including the field of computer-aided diagnosis (CAD) [8,9,10]. These technologies have demonstrated exceptional utility in enhancing X-ray-based CS classification, through their ability to autonomously identify and analyze CS-relevant features within X-ray images. The strengths of ML and DL lie in their capacity to manage and analyze large datasets, thereby improving the performance and generalizability of diagnostic algorithms. By minimizing the influence of human subjectivity and fatigue, these technologies ensure higher diagnostic precision and consistency. DL models, in particular, offer rapid analysis capabilities, essential in urgent care settings, and evolve over time through continuous training, further refining their accuracy. Consequently, ML and DL represent invaluable tools for healthcare professionals, providing critical support in achieving more accurate diagnoses and treatment decisions.

This research employs a ResNet-34 convolutional neural network (CNN) model to evaluate the presence of CS in patients, utilizing cervical spine X-ray imaging features. Notably, this study distinguishes itself by being the first to leverage multi-angle, multi-label data, enhancing the model’s accuracy significantly. The results confirm that the proposed deep learning neural network model achieves a diagnostic accuracy rate that is 21.4% higher than conventional manual diagnostic approaches [11], thereby offering a substantial improvement in the accuracy of CS diagnosis and potentially reducing the clinical workload.

In this work, our main contributions can be summarized as the following:

- Improved Patient Outcomes: The enhanced accuracy of the ResNet-34 CNN model, which is 21.4% higher than manual methods, means that it can more accurately diagnose conditions from cervical spine X-ray images. This leads to more accurate treatments being prescribed, improving patient outcomes.
- Efficiency in Diagnosis: The increased diagnostic accuracy reduces the reliance on manual methods, which can be time-consuming and prone to human error. This not only speeds up the diagnostic process but also reduces the workload of clinical staff, allowing them to focus on other critical tasks.
- Leveraging Multi-angle, Multi-label Data: This is the first model to leverage multi-angle, multi-label data, which provides a more comprehensive view of the patient’s condition. This can lead to the discovery of conditions that might be missed using traditional methods, further improving the accuracy of the diagnosis.

## 2. Proposed Method

### 2.1. Materials

This retrospective study meticulously compiled data from 595 patients treated at a rehabilitation research center from January 2021 to November 2022, resulting in a collection of 1371 anonymized images in line with established research protocols. The cohort consisted of 566 males and 805 females, with an average age of 46.4 ± 16.4 years, ensuring all selected medical images met the required diagnostic standards.

#### 2.1.1. Inclusion Criteria

For a case to be included in the study, patients had to meet the radiological diagnostic criteria for Cervical Spondylosis (CS). These criteria encompass a range of degenerative changes indicative of CS, including [12,13] degeneration observed in the intervertebral facet joints and vertebral costal joints, intervertebral disc degeneration, ligament degeneration, uncinate joint degeneration, osteoporosis, and scoliosis. Such comprehensive criteria ensure a robust identification of positive CS cases based on radiological evidence.

#### 2.1.2. Exclusion Criteria

The study excluded any patient showing degenerative changes in the cervical spine that could be attributed to congenital malformations, spinal cord injuries, tuberculosis, tumors, ankylosing spondylitis, or previous surgical interventions. Furthermore, X-ray images that did not meet the quality criteria due to artifacts, overexposure, underexposure, or any factor that could affect the clarity and accuracy of cervical spine imaging diagnosis were also excluded [14].

### 2.2. Experimental Design

This study strategically divided the 1371 collected image data into two sets: a test set (136 images) and a training-validation set (1235 images), following a 9:1 ratio. Each image was manually labeled by expert radiologists, noting the cervical spine X-ray projection position and CS classification outcomes.

**Figure 1:**
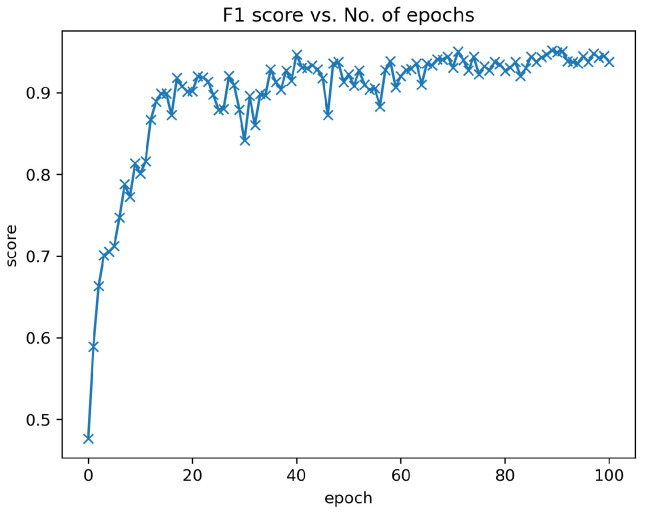
F1 score of ResNet-34 model from validation results. The abscissa is the F1-score of each validation from training the model, and the ordinate is the number of times the model is trained. We saved the final CNN from the validation with the best performance within 100 epochs.

Within the training-validation dataset, 80% (988 images) was randomly selected for the training of the ResNet-34 convolutional neural network (CNN) model [15], while the remaining 20% (247 images) constituted the validation set, which was strictly used for performance evaluation without influencing the training phase. The selection of the model for final experimentation was determined by the highest F1-score achieved on this validation set (Figure 1).

**Figure 2:**
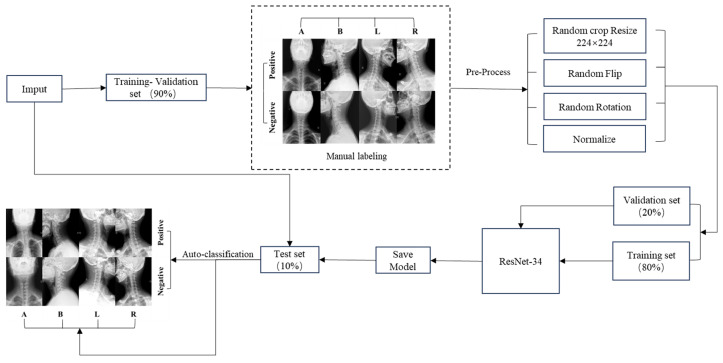
CNN model flow chart. We categorize the input data into a test set and a training-validation set. The data in the training-validation set undergo manual labeling, and the results are fed into the subsequent step. Subsequently, preprocessing is applied to the training-validation set data. Following preprocessing, the training set data are employed to train the ResNet-34 model, while the validation set data are utilized for selecting the final model. The ultimately saved model is then evaluated for performance using the test dataset.

The performance of the ResNet-34 CNN model in accurately classifying cervical spine X-ray projection positions and diagnosing CS was evaluated using the test set. Detailed methodologies and the results of this evaluation are elaborated upon in Section 3, accompanied by illustrative Figure 2 that maps out the process.

#### 2.2.1. Data Preprocessing

To prepare the X-ray images for CS research, each image underwent anonymization using the freely available RadiAnt DICOM Viewer 2020.2.3 software, effectively eliminating any patient identifiers. Data augmentation prepossessing includes randomly cropping the image to a size of 224 × 224 pixels, applying random flips, random rotations, and normalization.

#### 2.2.2. Data Labeling

For the purpose of image diagnosis, two expert radiologists, with eight and twenty-five years of specialized experience, respectively, independently assessed each patient’s set of images. These sets included anteroposterior, lateral, left oblique, and right oblique views, presented as separate, anonymized collections. The RadiAnt DICOM Viewer 2020.2.3 software facilitated the image evaluations. In instances of divergent assessments, the two experts engaged in discussions to reach a consensus. Cases without unanimous agreement were subsequently excluded from the analysis.

The process began with the initial labeling of the X-ray projection positions for each diagnostic image. Following this, CS was identified based on the distinct radiological diagnostic features observable in different positions. The labeled imaging characteristics for the various positions are outlined as follows:

Anteroposterior: (a) Degenerative changes in the intervertebral facet joints and vertebral costal joints, presenting as marginal osteophyte development, articular cartilage sclerosis, cystic degeneration, and narrowing of the joint space; (b) osteoporosis; (c) scoliosis.

Lateral: (a) Intervertebral disc degeneration, presenting as intervertebral space narrowing, the formation of Schmorl’s nodes, and the development of osteophytes along the vertebral body edges; (b) ligament degeneration, presenting as ligament thickening and calcification; (c) osteoporosis; (d) scoliosis, presenting as vertebral slippage and abnormal cervical curvature.

Left Oblique and Right Oblique: (a) Uncinate joint degeneration, presenting with osteophyte formation and intervertebral foramen narrowing; (b) osteoporosis.

#### 2.2.3. Deep Learning Model

The CNN stands as a prominent subcategory within the realm of DL algorithms and serves as the primary neural network architecture harnessed in the field of medical imaging [16]. Among various CNN architectures, ResNet stands out as a cutting-edge model for image classification, renowned for its efficacy and precision [17,18]. The adoption of ResNet-34 in this study is underpinned by its prior training on the ImageNet dataset, which is notably extensive and diverse, providing a solid foundation for image classification tasks.

A key innovation of ResNet-34 is its incorporation of residual learning, namely, a novel approach that emphasizes learning the residual or difference between the input and output. This strategy effectively addresses the perennial issues of vanishing and exploding gradients that often hamper the performance of traditional neural networks [19]. By fostering direct information flow across layers through cross-layer connections, ResNet-34 ensures the preservation of input data integrity throughout the network. This attribute not only streamlines the model’s mapping process but also diminishes training complexity.

The architectural hallmark of ResNet-34 is its assembly of residual blocks, which collectively forge a deep CNN. Such depth empowers the network to capture complex high-level features and patterns within the data, facilitating nuanced image analysis. Additionally, the modular design of ResNet-34’s residual blocks enhances the model’s versatility, making it apt for a wide array of computer vision tasks beyond image classification, including object detection and semantic segmentation. This versatility not only augments model reusability but also supports the application of transfer learning techniques, thereby broadening the scope of ResNet-34’s utility in diverse settings [20].

### 2.3. Statistical Analysis

Data analysis was conducted using SPSS 26. Measurement data were represented as $\bar{x}\pm s$ or $M(P_{25},P_{75})$. Counting data were presented as the number of cases and percentages (%).

Various evaluation metrics were employed to assess model performance. Accuracy was the primary metric used for evaluation, with additional criteria including sensitivity, precision, and F1-score (Table 1). The reliability and significance of these metrics were further scrutinized through $\chi^{2}$ testing, with a P > 0.01 indicating the absence of statistically significant differences within the dataset. For the execution of statistical analyses, the Scikit-learn library was employed, ensuring robust and reliable analysis [21].

In essence, classifiers that exhibit superior performance across the metrics of accuracy, sensitivity, precision, and F1-score are deemed more efficacious in the context of performance evaluation, providing a comprehensive measure of the model’s diagnostic capability.

## 3. Results

The dataset for this study comprises 1371 X-ray images, including 566 male and 805 female participants, with a mean age of (46.4 ± 16.4) years. Statistical analyses to assess the comparability of the training-validation and test sets with the overall population sample were performed using the $\chi^{2}$ test for categorical variables and the *T* test for continuous variables. These analyses covered parameters such as the number of images, gender distribution, age, and rates of positive diagnosis. The resulting P > 0.01, indicating the absence of statistically significant differences between the training-validation set, the test set, and the population sample. This demonstrates the external validity of the study findings; refer to Table 2.

### 3.1. Cervical Vertebra X-ray Projection Position Classification Results

Upon applying the neural network model to the test dataset, exemplary performance metrics were observed. The weighted average (WA), based on the proportions of images in anteroposterior (A), lateral (B), left oblique (L), and right oblique (R) positions, exhibited average accuracy, sensitivity, precision, and F1-score of 97.8%, 97.8%, 98.1%, and 97.8% respectively.

**Figure 3:**
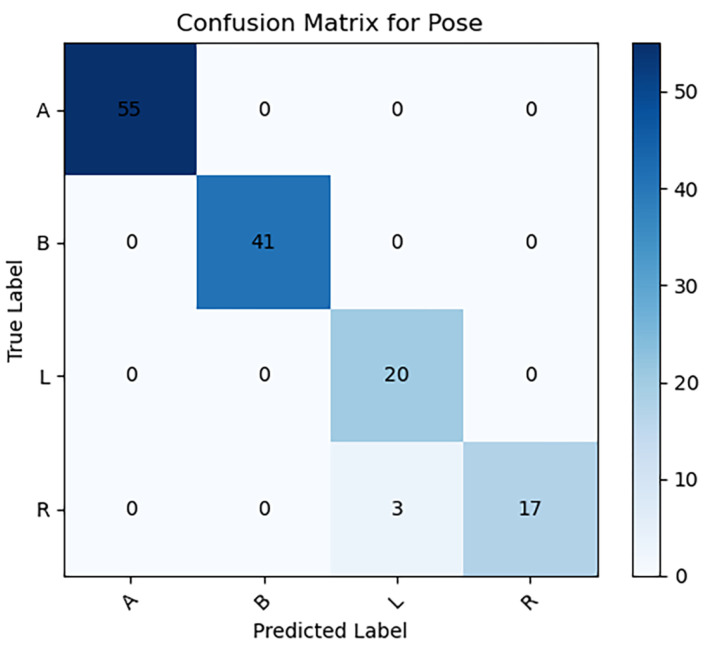
Confusion matrix for classification of cervical vertebrae X-ray projection position on test dataset.

Detailed analysis of the four specific imaging positions revealed optimal scores: positions A, B, and L demonstrated perfect accuracy and sensitivity (100.0%), underscoring the model’s exceptional efficacy. Positions A, B, and R recorded the highest precision (100.0%), and positions A and B achieved the top F1-score (100.0%). These indicators were validated through $\chi^{2}$ testing, confirming their statistical reliability as depicted in Figure 3 and Table 3).

### 3.2. CS Classification Results

When analyzing the test dataset with the neural network model, the performance metrics revealed are as follows: the weighted average (WA) results yield an average accuracy of 89.7%, sensitivity of 92.4%, precision of 91.1%, and F1-score of 91.2%.

**Figure 4:**
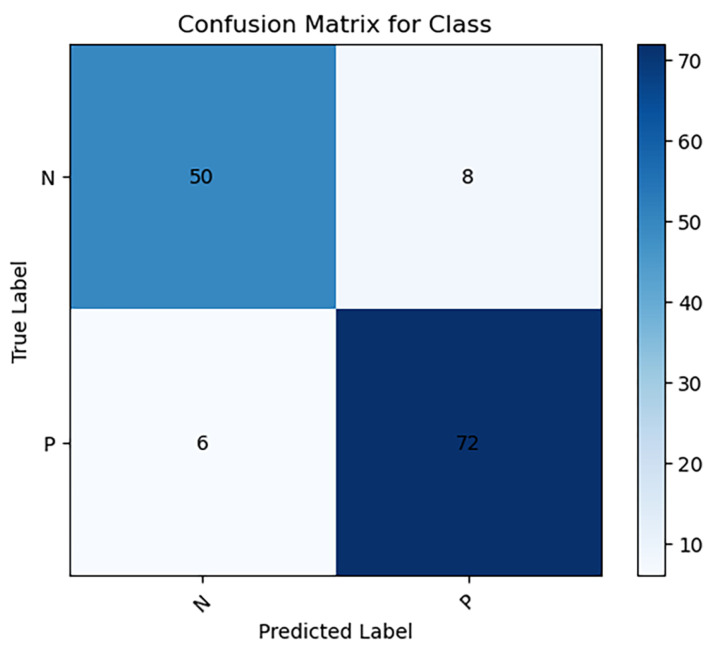
Confusion matrix for classification of CS on test dataset (P: positive, N: negative).

**Figure 5:**
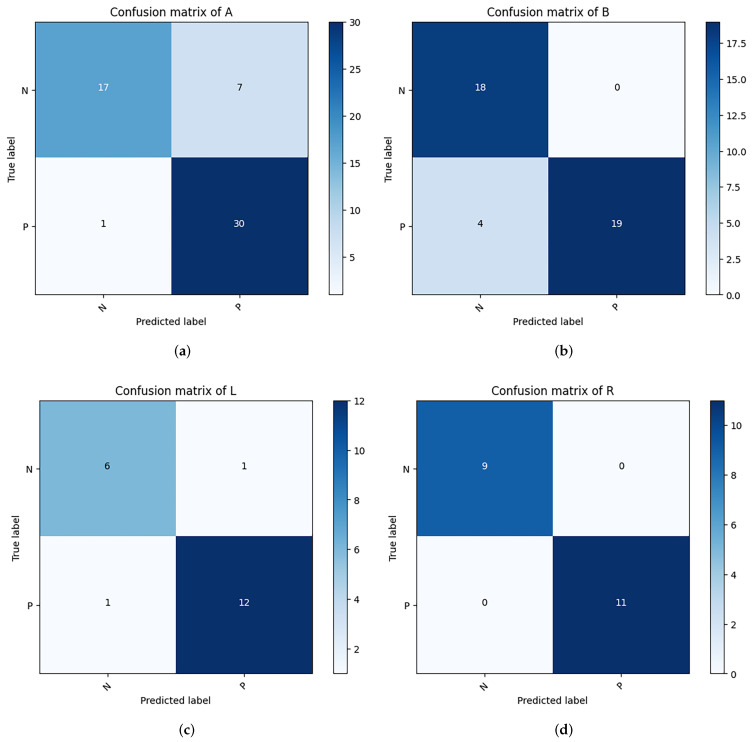
Confusion matrix for classification of CS from different positions. (**a**) is from anteroposterior positions; (**b**) is from lateral positions; (**c**) is from left oblique positions; (**d**) is from right oblique positions.

Further dissection of the Cervical Spondylosis (CS) classification outcomes across four distinct imaging positions indicates exceptional performance in the right oblique (R) position, with perfect scores of 100.0% in accuracy, sensitivity, precision, and F1-score. The lateral (B) position also recorded a precision rate of 100.0%. These performance metrics were subjected to $\chi^{2}$ testing, affirming that the CS classification accuracy across different positions does not significantly deviate from the overall observed accuracy. This analysis is visually supported in Figure 4 and Figure 5, and Table 4.

## 4. Discussion

Overcoming the subjective limitations inherent in manual X-ray diagnosis of Cervical Spondylosis (CS) and enhancing diagnostic accuracy have been consistent objectives within the research community. It has been indicated that the accuracy of manual X-ray diagnosis for CS is only 68.3% [11]. Conversely, the application of artificial intelligence (AI) offers a promising avenue for the prediction and diagnosis of CS [22]. Yu [23] developed a CS classification model using fuzzy computing theory, achieving an accuracy of 80.33%, thus demonstrating the potential of machine learning in classifying and processing various imaging features effectively.

This study extends these findings by integrating data from multiple cervical spine projection positions and conducting a comprehensive analysis of imaging features prevalent in CS diagnoses. The development of an innovative CS classification model based on ResNet-34 is reported, achieving a classification accuracy of 89.7%. The research is characterized by several advantages: (a) the integration of data from various X-ray projection positions, and (b) the exhaustive analysis of numerous imaging features.

### 4.1. Multi- X-ray Projection Position Imaging Data

Cervical spine X-ray imaging data from various positions, including anterior-posterior, lateral, left oblique, and right oblique, were consolidated. Identifying image features from different positions is crucial for model training, as each image feature has a primary observation position [24]. Initially, CS X-ray projection positions are identified to ensure the deep learning model learns the most important CS image features from these specific positions, aiding in precise feature localization and thus enhancing CS diagnosis accuracy.

In the field of AI-based CS prediction and diagnosis, the lateral view of the cervical spine remains a primary perspective due to the rich diagnostic information it provides and the minimal overlap and relatively simple anatomical structures of lateral cervical spine images, which simplify data processing [25]. Lee’s [25] study reported an accuracy of 87.1% for CS classification in lateral positions. A classification accuracy of 90.2% for CS in lateral positions was achieved by the model (Table 4).

Conversely, the anterior–posterior position of the cervical spine requires the classification of numerous CS imaging features, with added complexity due to the overlap of anatomical structures such as the calcification of thyroid cartilage, the hyoid bone, and the mandible. Limited research focuses on anterior–posterior positions of the cervical spine, mostly on segmentation-related studies. The model achieved an accuracy of 85.5% for CS classification in anterior-posterior positions (Table 4).

Oblique positions of the cervical spine present challenges due to overlapping anatomical structures and strict irradiation positioning requirements, leading to unstable feature extraction. However, their advantage lies in the fewer image features involved in the model, related primarily to hook joint degeneration and osteoporosis, simplifying data processing. Park [26] proposed a cervical spine oblique position classification model based on ResNet50, with an identification accuracy of 77% for foraminal stenosis. The model demonstrated an accuracy of 95.0% for CS classification in oblique positions, showing superior performance (Table 4).

To validate the performance consistency of the model in CS classification across different X-ray projection positions, indicators were compared between positions. Statistical results showed no significant difference in various indicators of CS classification between different positions, confirming the stable and consistent performance of the model (Table 5).

### 4.2. Multi-Label CNN Model

Current research predominantly examines single imaging features of Cervical Spondylosis (CS); however, the radiological diagnosis of CS necessitates a comprehensive analysis of multiple imaging features. This study aims to empower artificial intelligence to autonomously identify CS image features and perform image diagnosis. Unlike models that concentrate on a singular feature, this approach involves training the model with images that display various CS imaging features in different numbers and positions. A multi-label neural network model was adopted, designed to output the probability of each category, thus facilitating the flexible classification of multiple labels. Additionally, the efficiency of the algorithm and the generalization capability of the model were enhanced through the design of loss functions tailored for optimizing multi-task learning.

While analyses based on single image features have produced satisfactory outcomes as evidenced by studies such as Jebri’s [27] use of a machine learning-based random forest classifier model for detecting intervertebral space narrowing and osteophyte formation, Tamai’s [28] segmentation of cervical ligament calcification using the EfficientNet-B2 model, Fujimori’s [29] application of the EfficientNet-B4 model to identify cervical lordosis, and Chen’s [30] diagnosis of cervical spine scoliosis using the ResNet model. These contributions notwithstanding, CS cannot be adequately represented by single image features alone but rather as a constellation of multiple imaging features. Therefore, a comprehensive analysis of varied imaging features is more reflective of the clinical diagnostic requirements for CS. Given the complexities associated with processing data characterized by multiple imaging features, classifying multi-feature patterns remains a challenging endeavor. The high accuracy achieved in this study is primarily attributed to the neural network model’s extensive coverage of essential X-ray imaging features of CS, positioning this research at the forefront of clinical relevance.

**Figure 6:**
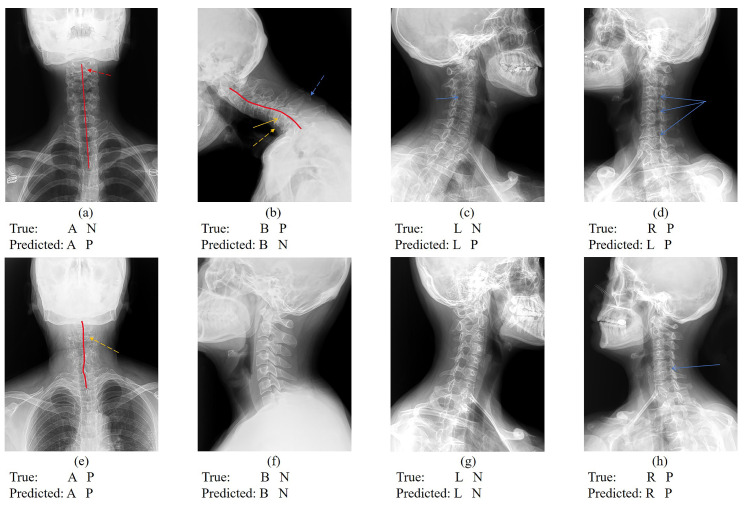
Cervical vertebra X-ray projection position classification and CS classification results. Figure (**a**–**c**) represent that the cervical spine position is correctly classified but CS is incorrectly classified. Figure (**d**) represents that the cervical spine position is classified incorrectly but CS is correctly classified. Figure (**e**–**h**) represent images with correct cervical vertebra position classification and CS classification. (“N” indicates CS is negative; “P” indicates CS is positive; the red line indicates the physiological curvature of the cervical spine; the red dotted arrow indicates the hyoid bone blocking the cervical vertebrae. the yellow arrow indicates the intervertebral space; the yellow dotted arrow indicates osteophyte formation; the blue dotted arrow indicates ligament calcification; and the blue arrow indicates the intervertebral foramen).

Despite the model’s exceptional performance in accuracy, sensitivity, precision, and F1-score, opportunities for enhancement remain. Notably, this retrospective study did not utilize standardized cervical spine imaging projection patterns. Variabilities such as hardware differences across devices, individual disparities resulting from X-ray imaging parameters (e.g., X-ray intensity, irradiation distance, and X-ray incidence angle), subjective imaging diagnostic criteria, and variations in patient positions complicate data processing (Figure 6). To mitigate overfitting associated with complex data, a novel model-processing method was employed. Parameters were saved not after every 100 training cycles but from the training sessions that exhibited the best performance within each set of 100 cycles. This approach, by minimizing the overfitting’s impact on model parameters, improved the model’s accuracy by 1.5% compared to traditional methods.

### 4.3. Comparative Performance Analysis

Table 6 showcases a comparative analysis of implementation accuracy among various deep convolutional neural network models. Notably, our study stands out with the ResNet34 model, achieving remarkable accuracy across all three views: anteroposterior (85.50%), lateral (90.20%), and oblique (95.00%). This comprehensive performance is unparalleled when compared to the other studies listed, which focus solely on the lateral view.

For instance, Yüksel’s [31] work with the VGG-16 model in 2022 achieved an accuracy of 93.90% in the lateral view, which is commendable but does not address the anteroposterior and oblique views. Similarly, other studies like those by Miura [32] and Tamai [28] using EfficientNetB4 and EfficientNetB2, respectively, show lower accuracies in the lateral view (86.00% and 88.00%) and do not provide solutions for the other views.

Moreover, Ogawa [33] and Lee’s [25] research with CNN models in 2022 also focus on the lateral view, with accuracies of 90.00% and 87.10% respectively, which are surpassed by our ResNet34’s performance. Park’s [26] study using ResNet50 is the only other work addressing a different view, the oblique but with a significantly lower accuracy of 77.00%.

Our method’s superiority is evident not only in the highest accuracy achieved in the lateral view but also in its versatility and robustness, demonstrated by its performance in the anteroposterior and oblique views. This indicates a significant advancement in the field, suggesting that our ResNet34 model can be a more reliable and comprehensive tool for image analysis in various orientations. The breadth and depth of accuracy in our study underscore its potential as the leading methodology in deep convolutional neural network research for image classification.

## 5. Conclusions

This study underscores the escalating prevalence of Cervical Spondylosis (CS) amidst evolving societal dynamics and lifestyle modifications, illuminating the consequential surge in the necessity for efficient and accurate CS diagnostic methodologies. The paramount importance of early intervention in the management and prognosis of CS is highlighted against the backdrop of the current limitations encountered in the diagnostic accuracy of CS via X-ray imaging. These limitations are primarily attributed to the non-distinct nature of X-ray imaging features and the substantial reliance on the diagnostic acumen of medical practitioners.

The deployment of the proposed neural network model represents a significant stride toward mitigating these challenges. Model selection is very important, and model optimization algorithms [34] are also the direction to improve accuracy in the future. By harnessing advanced machine learning techniques, the model demonstrates a robust capacity to accurately identify CS from X-ray images, thus offering a critical tool to support clinicians, particularly those at the nascent stages of their careers or those with limited diagnostic experience. Consequently, this model stands to substantially reduce the incidence of diagnostic errors, such as misdiagnoses or missed diagnoses, and to alleviate the workload burden on healthcare professionals.

Furthermore, the findings from this study lay a foundation for future research endeavors aimed at refining AI-driven diagnostic models, with the potential to enhance the precision and reliability of CS detection and classification. It opens avenues for the integration of such models into clinical workflows, thereby augmenting the diagnostic process with a level of accuracy and efficiency that aligns closely with the nuanced demands of contemporary medical practice.

In conclusion, the development and application of the neural network model for CS diagnosis not only advance the field of medical diagnostics but also promise significant improvements in patient care outcomes. By addressing the challenges associated with the current diagnostic modalities for CS, this study contributes to the broader narrative of leveraging artificial intelligence to enrich healthcare delivery, underscored by a commitment to accuracy, efficiency, and accessibility.

## Figures and Tables

**Table 1:** Calculation formula for evaluation indicators.

Indicators	Definition and Calculation Formula
Accuracy	(True Positive + True Negative) / (Population Sample)
Sensitivity	True Positive / (True Positive + False Negative)
Precision	True Positive / (True Positive + False Positive)
F1 Score	2 × (Precision × Sensitivity) / (Precision + Sensitivity)

**Table 2:** Demographic information.

	Population	Training-Verification Set	Statistics	P	Test Set	Statistics	P
No. of images	1371	1235	0.002	>0.999	136	0.036	0.998
A ^1^	565	509	/	/	55	/	/
B ^2^	410	370	/	/	41	/	/
L ^3^	196	176	/	/	20	/	/
R ^4^	200	180	/	/	20	/	/
Gender	Female 805(58.7%)	Female 724(58.6%)	0.002	0.962	Female 81(59.6%)	0.036	0.849
Age	46.4 ± 16.4	46.2 ± 16.0	−0.256	0.798	47.8 ± 20.3	0.976	0.329
Positive	57.8%	57.7%	<0.001	0.985	58.1%	0.005	0.943

The test method for number of images, gender and positivity rate indicators is the $\chi^{2}$ test. The test method for age indicators is the *T* test. ^1^ represents the anteroposterior position of the cervical spine. ^2^ represents the lateral position of the cervical spine. ^3^ represents the left oblique position of the cervical spine. ^4^ represents the right oblique position of the cervical spine.

**Table 3:** Statistical table for classification of cervical vertebrae X-ray projection position.

	Number	Accuracy	Statistics	P	Sensitivity	Statistics	P	Precision	Statistics	P	F1 Score	Statistics	P
A	55	100.0%	1.233	0.267	100.0%	1.233	0.267	100.0%	1.233	0.267	100.0%	1.233	0.267
B	41	100.0%	0.920	0.337	100.0%	0.920	0.337	100.0%	0.920	0.337	100.0%	0.920	0.337
L	20	100.0%	0.450	0.502	100.0%	0.450	0.502	87.0%	7.717	0.005	93.0%	0.545	0.460
R	20	85.0%	7.717	0.005	85.0%	7.717	0.005	100.0%	0.450	0.502	91.9%	3.414	0.065
O ^1^	40	92.5%	2.631	0.105	92.5%	2.631	0.105	93.5%	2.631	0.105	92.5%	2.631	0.105
WA ^2^	136	97.8%	/	/	97.8%	/	/	98.1%	/	/	97.8%	/	/

A, B, L, R and O are independently contrasted with the WA. The test method used $\chi^{2}$ test; if P > 0.01, it indicated no statistically significant differences in the data. ^1^ represents the set of right and left oblique positions of the cervical spine. ^2^ represents the weighted average according to the proportion of A, B, L, R.

**Table 4:** Statistical table for classification of CS.

	Number	Accuracy	Statistics	P	Sensitivity	Statistics	P	Precision	Statistics	P	F1 Score	Statistics	P
A	55	85.5%	0.695	0.405	96.8%	0.919	0.338	81.1%	3.365	0.067	88.2%	0.200	0.655
B	41	90.2%	0.010	0.920	82.6%	3.428	0.064	100.0%	3.881	0.049	90.5%	0.033	0.855
L	20	90.0%	0.002	0.968	92.3%	0.172	0.678	92.3%	0.030	0.864	92.3%	0.030	0.864
R	20	100.0%	2.262	0.133	100.0%	1.571	0.210	100.0%	1.912	0.167	100.0%	1.912	0.167
O	40	95.0%	1.048	0.306	96.2%	0.269	0.604	96.2%	0.617	0.432	96.2%	0.617	0.432
WA	136	89.7%	/	/	92.4%	/	/	91.1%	/	/	91.2%	/	/

**Table 5:** Performance comparison of deep learning models for CS classification at different projection positions.

Compare	Accuracy		Sensitivity		Precision		F1 Score	
	Statistics	P	Statistics	P	Statistics	P	Statistics	P
A–B	0.493	0.483	4.992	0.025	6.487	0.011	0.033	0.855
A–O	2.240	0.134	0.107	0.744	3.646	0.056	0.019	0.306
B–O	0.668	0.414	2.988	0.084	2.102	0.147	0.668	0.414
L–R	2.105	0.147	2.105	0.147	2.105	0.147	2.105	0.147
A–L	0.262	0.609	1.176	0.278	0.731	0.393	0.013	0.910
A–R	3.256	0.071	0.747	0.387	4.196	0.041	2.372	0.124
B–L	0.001	0.976	0.535	0.465	4.239	0.040	0.001	0.976
B–R	2.088	0.148	3.875	0.050	<0.001	>0.999	2.088	0.148

A, B, L, R, and O were compared pairwise. The test method used $\chi^{2}$ test; if P > 0.01, it indicated no statistically significant differences in the data. Dash (–) represents the comparison between two projected positions.

**Table 6:** Implementation accuracy comparison with previous research works using deep convolutional neural network.

Author	Year	Model	Accuracy		
			Anteroposterior	Lateral	Oblique
Yüksel et al. [31]	2022	VGG-16	-	93.90%	-
Miura et al. [32]	2021	EfficientNetB4	-	86.00%	-
Tamai et al. [28]	2022	EfficientNetB2	-	88.00%	-
Ogawa et al. [33]	2022	CNN	-	90.00%	-
Lee et al. [25]	2022	CNN	-	87.10%	-
Park et al. [26]	2022	ResNet50	-	-	77.00%
Our study		ResNet34	85.50%	90.20%	95.00%

## Data Availability

The datasets generated and/or analyzed during the current study are available from the corresponding author upon reasonable request.

## Abbreviations

The following abbreviations are used in this paper:

CS	cervical spondylosis
AI	artificial intelligence
ML	machine learning
DL	deep learning
CNN	convolutional neural network
A	the anteroposterior position of the cervical spine
B	the lateral position of the cervical spine
L	the left oblique position of the cervical spine
R	the right oblique position of the cervical spine
O	the set of right and left oblique positions of the cervical spine
WA	the weighted average based on the proportions of images in A, B, L, and R positions
